# Amygdalin Promotes Fracture Healing through TGF-*β*/Smad Signaling in Mesenchymal Stem Cells

**DOI:** 10.1155/2020/8811963

**Published:** 2020-09-07

**Authors:** Jun Ying, Qinwen Ge, Songfeng Hu, Cheng Luo, Fengyi Lu, Yikang Yu, Taotao Xu, Shuaijie Lv, Lei Zhang, Jie Shen, Di Chen, Peijian Tong, Luwei Xiao, Ju Li, Hongting Jin, Pinger Wang

**Affiliations:** ^1^Department of Orthopaedic Surgery, The First Affiliated Hospital of Zhejiang Chinese Medical University, Hangzhou, 310006 Zhejiang Province, China; ^2^Institute of Orthopaedics and Traumatology, The First Affiliated Hospital of Zhejiang Chinese Medical University, Hangzhou, 310053 Zhejiang Province, China; ^3^First Clinical College of Zhejiang Chinese Medical University, Hangzhou, 310053 Zhejiang Province, China; ^4^Department of Orthopaedics, Shaoxing Hospital of Traditional Chinese Medicine, Affiliated with Zhejiang Chinese Medical University, Shaoxing, 312000 Zhejiang Province, China; ^5^Department of Orthopaedic Surgery, Fuyang Orthopaedics and Traumatology Affiliated Hospital of Zhejiang Chinese Medical University, Hangzhou, Zhejiang, China; ^6^Department of Orthopedics, Xiaoshan District Hospital of Traditional Chinese Medicine of Hangzhou, Hangzhou, 311201 Zhejiang Province, China; ^7^Department of Orthopaedic Surgery, School of Medicine, Washington University, St. Louis, MO 63110, USA; ^8^Research Center for Human Tissues and Organs Degeneration, Shenzhen Institutes of Advanced Technology, Chinese Academy of Sciences, Shenzhen 518055, China

## Abstract

Chondrogenesis and subsequent osteogenesis of mesenchymal stem cells (MSCs) and angiogenesis at injured sites are crucial for bone fracture healing. Amygdalin, a cyanogenic glycoside compound derived from bitter apricot kernel, has been reported to inhibit IL-1*β*-induced chondrocyte degeneration and to stimulate blood circulation, suggesting a promising role of amygdalin in fracture healing. In this study, tibial fractures in C57BL/6 mice were treated with amygdalin. Fracture calluses were then harvested and subjected to radiographic, histological, and biomechanical testing, as well as angiography and gene expression analyses to evaluate fracture healing. The results showed that amygdalin treatment promoted bone fracture healing. Further experiments using MSC-specific transforming growth factor- (TGF-) *β* receptor 2 conditional knockout (KO) mice (*Tgfbr2^Gli1-Cre^*) and C3H10 T1/2 murine mesenchymal progenitor cells showed that this effect was mediated through TGF-*β*/Smad signaling. We conclude that amygdalin could be used as an alternative treatment for bone fractures.

## 1. Introduction

Bone fractures, mainly caused by traumatic incidents and medical conditions, including osteoporosis, are a growing global health burden currently affecting millions of people [1, 2]. Even with treatment, fractures are associated with great economic burden, loss of independence, and high rates of morbidity and mortality [3]. In clinical practice, fractures can be stabilized using various fixation methods, such as braces and internal, external, and intramedullary fixation [4–6]. However, fracture nonunion is observed in 5-10% of patients annually [7]. In addition, bedfast patients receiving conservative treatment have higher risks of pressure ulcers, hypostatic pneumonia, lower extremity venous thromboembolism, and death.

Bone fracture healing is a complicated biological process that involves specific regenerative patterns and diverse gene expression changes [8, 9]. Secondary healing is the most common form of bone healing, which normally includes endochondral and intramembranous bone healing that can be separated into three consecutive stages: inflammation, bone repair, and remodeling [9]. The inflammatory phase is characterized by the formation of a hematoma and the immediate release of soluble inflammatory mediators that induce immune cell infiltration [10].

Mesenchymal stem cells (MSCs) can migrate to the fracture site and differentiate into chondrocytes, which form a cartilaginous soft callus. This primary callus is subsequently surrounded by new bone material produced by osteoblasts in the perichondrium and permeated with blood vessels. The cartilaginous callus then undergoes remodeling to replace the cartilage with new bone. Thus, the promotion of chondrogenesis/osteogenesis and angiogenesis can accelerate fracture healing. The origin of MSCs is not fully understood, but the recruitment, proliferation, and differentiation of these cells are indispensable for fracture healing [11]. Successful bone repair and remodeling are also dependent on an adequate blood supply and revascularization of the injured area [12].

Amygdalin (D-mandelonitrile-*β*-gentiobioside) is derived from bitter apricot kernel and has been used clinically for the treatment of asthma, aplastic anemia, tumors, and alloxan-induced diabetes [13–15]. Amygdalin has also been reported to inhibit IL-1*β*-induced chondrocyte degeneration in endplates and to both improve microcirculation and relieve blood stasis [16, 17]. Amygdalin has been implicated in the regulation of transforming growth factor- (TGF-) *β*/Smad signaling [18], which has a fundamental regulatory function for bone homeostasis [19, 20]. Therefore, we hypothesized that amygdalin can promote bone fracture healing through TGF-*β*/Smad signaling.

## 2. Materials and Methods

### 2.1. Preparation and Administration of Amygdalin

**Figure 1:**
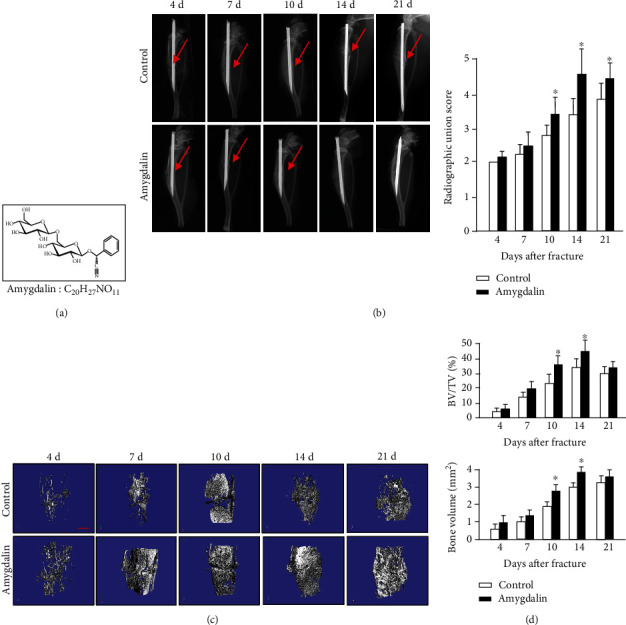
Amygdalin accelerated bone fracture healing in mice. (a) The chemical structure of amygdalin. (b) X-ray images and radiographic union scores showed clear fracture lines at days 4-21 in mice of the control group, but only at days 4-10 in the amygdalin-treated group. Red arrows point to clear fracture lines. (c). *μ*CT three-dimensional images of fractured tibiae from both groups at days 4, 7, 10, 14, and 21. Scale bar = 500 *μ*m. (d) *μ*CT analysis of BV/TV. Data are presented as mean ± standard deviation (SD). ∗*P* < 0.05, *n* = 6.

Amygdalin (CAS NO. 29883-15-6, purity ≥ 97.0%, Sigma, St. Louis, MO, USA) (Figure 1(a)) used in this study was purchased from the National Institutes for Food and Drug Control of China and dissolved in sterile normal saline (NS) (0.05 mg/mL). In the amygdalin-treated group, mice were intraperitoneally injected with 0.5 mg/kg of amygdalin daily from day 1 postoperation. Mice in the control group were intraperitoneally injected with an equal amount of phosphate-buffered saline (PBS).

### 2.2. Experimental Animals

This study was approved by the Animal Experimentation Ethics Committee of Zhejiang Chinese Medical University. The animal center of the Zhejiang Chinese Medical University provided C57BL/6 mice (SCXK, Shanghai, 2012-0002). *Gli1-CreER* transgenic mice can efficiently target MSCs by injecting tamoxifen (TM) to induce the formation of Cre (CreERT2) from the endogenous Gli1 locus [21]. The *Gli1-CreER* transgenic mice were crossed with *Tgfβr2^flox/flox^* mice [22] to specifically knock out the TGF-*β* receptor 2 (*Tgfbr2*) in MSCs. Tamoxifen was administered (5 mg, dissolved in corn oil) once daily for 5 consecutive days by intraperitoneal injection.

A DNA extraction kit (Sigma, USA) was used to determine mouse genotyping. The sequences of primers used for genotyping were as follows: Cre, Fw: 5′-ATT GCT GTC ACT TGG TCG TGGC-3′; Rv: 5′-GAA AAT GCT TCT GTC CGT TTGC-3′; Tgfbr2 loxP, Fw: 5′-TAA ACA AGG TCC GGA GCC CA-3′; Rv: 5′-ACT TCT GCA AGA GGT CCC CT-3′ (wild-type, 420-base-pair PCR product; homozygotic type, 540-base-pair PCR product). Cre-negative littermates were used as controls.

### 2.3. Cell Culture

C3H10 T1/2 murine mesenchymal progenitor cells (ATCC, Manassas, VA, USA) were cultured in Dulbecco's modified Eagle medium (DMEM) (Hyclone, Logan, Utah, USA) supplemented with 10% fetal bovine serum (FBS), 100 U/mL penicillin and 100 mg/mL streptomycin (Gibco, Grand Island, NY, USA) in a 5% CO_2_ humidified incubator at 37°C. After reaching 80% confluence, cells were collected and divided into the control group, the TGF-*β*1 group (treated with 10 ng/mL recombinant TGF-*β*1), the amygdalin group (treated with 10 *μ*M amygdalin), the TGF-*β*1 plus TGF-*β*/Smad pathway inhibitor-SB525334 group (treated with 10 ng/mL recombinant TGF-*β*1 and 10 *μ*M SB525334), the amygdalin plus SB525334 group (treated with 10 *μ*M amygdalin and 10 *μ*M SB525334), and the SB525334 group (treated with 10 *μ*M SB525334). After 4 hours of treatment, the cells were collected, and changes in the levels of phosphorylated-Smad2/3 were analyzed. In addition, relative chondrogenic (Col2a1, Sox9) gene expressions were analyzed following three days in culture.

### 2.4. Tibial Fracture Model

Right transverse tibial fractures were performed and fixed with an intramedullary needle [23]. Ten-week-old male C57BL/6, *Tgfβr2^f/f^*, and *Tgfβr2^Gli1Cre^* mice were anesthetized with ketamine (60 mg/kg) by intraperitoneal injection. After local disinfection, a 1.0 cm long cut in the anteromedial skin of the tibia was created. A 26-gauge syringe needle was then inserted into the bone marrow cavity through the tibial plateau at the medial of patellar ligament. The needle was then removed, and the tibia was transected using a No.11 surgical blade. The 26-gauge syringe needle was reinserted into tibia to simulate intramedullary fixation. A 5-0 silk suture was selected to close the incision, and buprenorphine administration (in drinking water) was used to reduce pain during the first three days following surgery. Immediately following surgery, X-ray tests (Carestream, FX Pro, USA) were performed on the right lower limb in the anterior-posterior and lateral directions to confirm the correctness of osteotomy and the alignment of the bone. The mice were then divided into groups for analysis: C57BL/6 mice treated with amygdalin or PBS, and *Tgfβr2^Gli1Cre^* and Cre-negative controls both treated with amygdalin.

### 2.5. X-Ray and *μ*CT Analyses

At days 4, 7, 10, 14, and 21 postsurgery, mice were euthanized, and tibiae were collected for X-ray and microcomputed tomography (*μ*CT) (Skyscan 1176; Bruker *μ*CT, Kontich, Belgium) analyses to examine fracture healing quality. At each time point, harvested specimens were scanned using *μ*CT at 8.73-micron isotropic resolution, with settings of 42 kV and 555 *μ*A and 786 ms integration time. Followed by radiographic union scale (RUST) in the tibial fracture score system, semiquantitative analyses of the X-ray were performed. Callus bone volume (BV) and callus mineralized volume fraction (BV/TV) (%) parameters were then measured.

### 2.6. Biomechanical Testing

Full-length tibiae were harvested and removed from their surrounding soft tissues (*n* = 6 at days 4, 7, 10, 14, and 21). Both ends of each tibia were then fixed in bone cement to ensure that the fracture site was exposed. Specimens were installed on an Endura Tec TestBench TM system (200 N mm torque cell; Bose Corporation, Minnetonka, MN, USA) to apply torsion at a rate of 1°/s until failure to determine the modulus of elasticity and maximum loading of the fracture callus.

### 2.7. Histology and Histomorphometry

After three days of fixation with 10% normal buffered formalin, the bone samples were decalcified in 14% ethylenediaminetetraacetic acid (EDTA) for two weeks. The decalcified bones were dehydrated and embedded in paraffin and sectioned (3 *μ*m). Alcian blue/H&E (ABH) staining and tartrate-resistant acid phosphatase (TRAP) staining were performed. Quantitative histomorphometry was performed on the ABH- and TRAP-stained sections using the ImageJ 1.46r software (Wayne Rasband, National Institute of Health, USA). The cartilage area per periosteal callus area (%, Cg.Ar/http://Ps.Cl.Ar) and the mineralized area per periosteal callus area (%, Md.Ar/http://Ps.Cl.Ar) were measured as described previously [24]. Immunohistochemistry (IHC) was performed on sections using high-pressure heating in pH 6.0 sodium citrate solution for 2 minutes to achieve antigen retrieval. The tissues were then incubated with a rabbit anti-mouse anti-p-Smad2 antibody (Abcam, ab188334, 1 : 200), anti-pSmad1/5/8 antibody (Millipore, AB3848, 1 : 200), and anti-CD31 antibody (Arigo, ARG52748, 1 : 100) overnight at 4°C. PBS was incubated on negative group slides. The following day, a biotinylated anti-rabbit secondary antibody and an enzyme conjugate (Histostain-Plus Kit, Invitrogen, USA) were separately incubated on sections. IHC signals were developed using DAB chromogen (Histostain-Plus Kit) and counterstained for nuclei using Tacha's CAT Hematoxylin.

### 2.8. Quantitative Real-Time Polymerase Chain Reaction

The callus was cut with a range of 2 mm around the fracture line. Total RNA was extracted from the callus and three duplicated wells of C3H10 T1/2 cells in the third passage by using the RNeasy kit (Qiagen, Germany) following the manufacturer's instructions. Then, complementary DNA (cDNA) was synthesized using a cDNA reverse transcription kit (Takara, Otsu, Japan). Quantitative real-time polymerase chain reaction (qRT-PCR) was performed using the SYBR Premix EX Taq™ kit (Takara) according to the manufacturer's instructions. Primer sequences for *Sox9*, *Col2a1*, *Col10a1*, *Osteocalcin*, *Runx2*, vascular endothelial growth factor (*Vegf*), and *β*-actin are shown in Table 1.

### 2.9. Western Blot Analysis

Three duplicated wells of C3H10T1/2 cells at passage 3 were prepared for protein extraction. Cell lysates were extracted using a modified radioimmunoprecipitation assay (RIPA) lysis buffer that contained 1 mM phenylmethylsulfonyl fluoride (PMSF) and a protease inhibitor cocktail (Cell Signaling Technology, USA). Following centrifugation at 12000g for 30 minutes, the supernatant was collected to detect the total protein concentrations using a BCA Protein Assay kit (Thermo Scientific, USA). Protein extracts were boiled for 5 minutes in loading buffer. Then, 40 *μ*g protein was loaded and electrophoresed on a sodium dodecyl sulfate polyacrylamide gel electrophoresis (SDS-PAGE) gel and transferred to a polyvinylidene difluoride (PVDF) membrane. The membrane was blocked in a 5% skim milk solution and incubated with primary antibodies overnight at 4°C. The primary antibodies were *β*-actin (Sigma, A1978), Smad2/3 (Cell Signaling Technology, #8685), and phosphorylated-Smad2/3 (Cell Signaling Technology, #8828). The membrane was then incubated with a fluorescent secondary antibody (LI-COR, 926-32212) (1/1000), for 1 hour at room temperature (Vector Laboratories, Burlingame, VT, USA). The blots were visualized using a LI-COR Odyssey® scanner (LI-COR Biosciences, USA). The relative density of the p-Smad2/3 bands was normalized to their corresponding actin bands.

### 2.10. Wound Scratch Test and Transwell Cell Migration

C3H10 T1/2 cells were seeded in six-well plates and cultured with DMEM/F12 supplemented with 5% FBS, 1 × 10^5^ units/L penicillin, and 100 g/L streptomycin. After reaching 80%-90% confluence, a scratch of ~0.5 mm was made using a sterile pipette tip and then washed with PBS to clear cellular debris. After injury, the cells were cultured with low glucose DMEM/F12 containing 5% (*v*/*v*) FBS with 0, 10 *μ*M amygdalin, 10 ng/mL TGF*β*1, 10 *μ*M amygdalin plus 10 *μ*M SB525334, 10 ng/mL TGF*β*1 plus 10 *μ*M SB525334, or 10 *μ*M SB525334. The migrations of C3H10 T1/2 cells into the scratch were photographed using an inverted microscope (Olympus, Tokyo, Japan) at 24 hours post insult.

Transwell assays were performed using 8 *μ*m-pore Transwell polycarbonate membranes (Corning Inc., Corning, New York, USA). 1.0 × 10^4^ cells were seeded with 200 *μ*L DMEM/F12 containing 2% FBS in the upper chamber, and in the lower chamber, 500 *μ*L DMEM/F12 containing 2% FBS with 0, 10 *μ*M amygdalin, 10 ng/mL TGF*β*1, 10 *μ*M amygdalin plus 10 *μ*M SB525334, 10 ng/mL TGF*β*1 plus 10 *μ*M SB525334, or 10 *μ*M SB525334. After incubating for 48 h, the migratory cells were fixed with 4% paraformaldehyde for 15 min and stained with crystal violet. Three random images were taken in the fields.

### 2.11. *In Vivo* Angiography

After injecting a mouse with ketamine (60 mg/kg), the right atrium was perforated, and 10 mL PBS/heparin sodium was injected through the apex of the left ventricle. Subsequently, 10 mL of 4% paraformaldehyde was perfused using the same method. Finally, 10 mL MICROFIL was injected with more pressure due to its viscosity and conserved overnight at 4°C. Muscles around the bone callus were removed to eliminate the influence of muscle blood vessels. *μ*CT scanning was first used to locate the callus surrounding the fracture. After decalcification of the harvested bone samples for 3 days, the samples were scanned again, using the same volume of interest (VOI). The analysis area included 400 slices by considering the fracture line as the midline. The CTAn Software (Skyscan, Version 1.16) was used to build three-dimensional (3D) models and to measure relative vessel volume.

### 2.12. Statistical Analysis

All data were obtained from 6 individual mice in every experiment and 3 duplicated wells of C3H10 T1/2 cells. Values are showed as the mean. Two-way ANOVA followed by the Tukey-Kramer posttest (multiple groups) and unpaired Student's *t*-test (two groups) were used for statistical analyses. The SPSS 17.0 software was used to perform the statistical tests. *P* < 0.05 was considered significant.

## 3. Results

### 3.1. Amygdalin Facilitates Bone Fracture Healing in Mice

To assess the effects of amygdalin on fracture healing, mice were treated with PBS or amygdalin (0.5 mg/kg/day). X-ray results showed that treatment with amygdalin promoted earlier formation of bone callus (Figure 1(b)). In the amygdalin group, a clear callus profile could be observed at day 10, and the callus became more radiopaque at day 14 postfracture. In contrast, the callus was not apparent in the control group until day 14 postfracture.

In addition, 3D reconstructions of *μ*CT images showed that obvious fracture gaps could be observed in the control group at days 4, 7, 10, and 14, and newly formed bone had not fully bridged the gaps until day 21 (Figure 1(c), left). In contrast, in the amygdalin-treated group, the fracture gap appeared indistinct at day 14, indicating a higher proportion of mineralized bone in the callus (Figure 1(c), right). *μ*CT analysis revealed that the bone volume (%, BV/TV) in the amygdalin group was significantly increased compared to the control group at days 10 and 14 (Figure 1(d)), consistent with the radiographic results.

**Figure 2:**
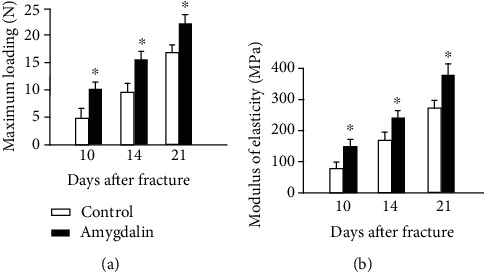
Biomechanical testing was performed in bone samples harvested at days 10, 14, and 21 after surgery. (a) Maximum loading and (b) modulus of elasticity were measured in the amygdalin-treated and control groups. Data are presented as mean ± standard deviation (SD). ∗*P* < 0.05, *n* = 6.

Furthermore, biomechanical testing showed that the modulus of elasticity and maximum loading increased significantly in tibia samples at days 10, 14, and 21 in the amygdalin-treated group compared to the PBS control group (*P* < 0.05). These data demonstrated that bone mechanical strength has been improved by amygdalin treatment (Figure 2).

**Figure 3:**
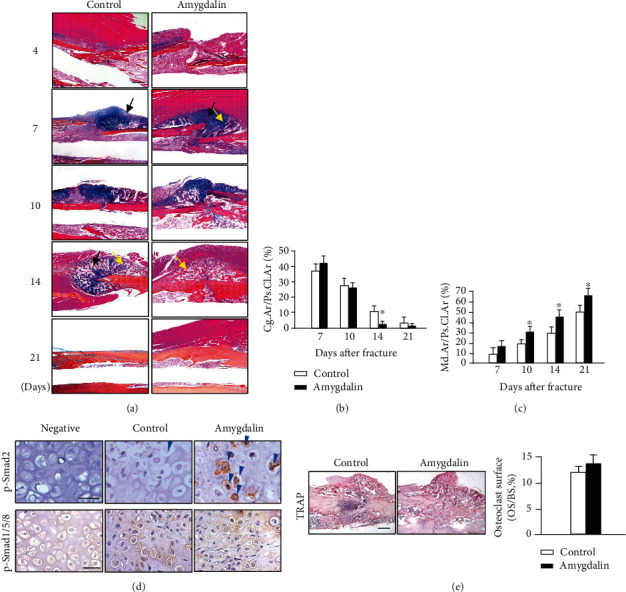
Amygdalin accelerated formation of woven bone. (a) Histological sections of the bone fracture with Hematoxylin and Eosin (H&E) as well as Alcian blue staining. Black arrows represent the cartilage area; yellow arrows highlight the woven bone area. Scale bar = 200 *μ*m. (b) The cartilage area (blue) of callus tissues (%, Cg.Ar/http://Ps.Cl.Ar) was quantified at days 7, 10, 14, and 21 postfracture in the control and amygdalin-treated groups. (c) Newly formed woven bone in callus tissues (%, Md.Ar/http://Ps.Cl.Ar) was quantified at days 7, 10, 14, and 21 postfracture in the control and amygdalin-treated groups. (d) Phospho-Smad2 and phospho-Smad1/5/8 protein expressions were assessed by immunohistochemistry in control and amygdalin-treated groups at days 7 and 10, separately. Blue arrows: p-Smad2 positive cells. Scale bar = 50 *μ*m. Data are presented as mean ± standard deviation (SD). (e) Tartrate-resistant acid phosphatase (TRAP) staining and quantification of osteoclast surface per bone surface (Oc. S./BS) of sections from control and amygdalin-treated groups at day 14 after surgery. Scale bar = 100 *μ*m. ∗*P* < 0.05, *n* = 6.

Because radiographic evaluation cannot detect changes in cartilage and soft tissues, Alcian blue/Hematoxylin/Orange G (ABH/OG) staining and histomorphometric analysis of the fractured bone were performed. The cartilage area (%, Cg.Ar/http://Ps.Cl.Ar) was significantly reduced by day 14 postfracture, and the mineralized bone area was significantly increased (%, Md.Ar/http://Ps.Cl.Ar) at days 10-21 postfracture in amygdalin-treated mice compared to the control. At day 21, the cartilage was no longer detectable in either group (Figures 3(a)–3(c)). In addition, phospho-Smad2 and phospho-Smad1/5/8 IHC was performed to observe activation of TGF-*β*/Smad and BMP2 signaling during fracture healing. As shown in Figure 3(d), amygdalin treatment could increase phospho-Smad2 expression at day 7 but have no obvious effect on phospho- Smad1/5/8 expression at day 10. TRAP staining was also performed in the fracture callus at day 14. No significant changes in osteoclast numbers were found after amygdalin treatment at day 14 (Figure 3(e)).

**Figure 4:**
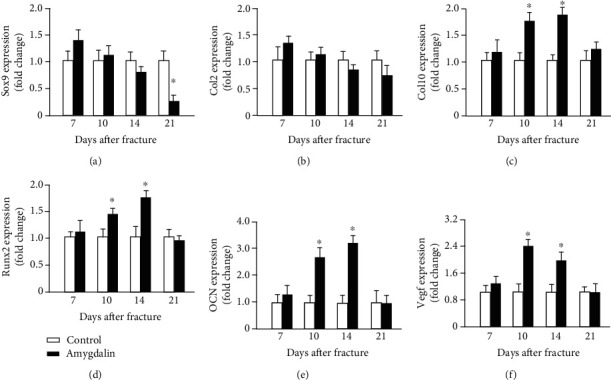
Amygdalin upregulated expression of chondrogenesis-, osteogenesis-, and angiogenesis-related genes in the callus. (a–f) Expressions of *Sox9*, *Col2a1*, *Col10a1*, *Runx2*, *Ocn*, and *Vegf* were measured by quantitative real-time polymerase chain reaction (PCR) between control and amygdalin groups at days 7, 10, 14, and 21 postfracture. Data are the relative expression versus housekeeping gene *β*-actin and are presented as mean ± standard deviation (SD). ∗*P* < 0.05, *n* = 6.

We also measured the expression of marker genes related to bone fracture healing. The gene expression of early chondrogenesis, such as *Sox9* and *Col2a1*, was similar in amygdalin-treated and control mice during fracture healing, except that *Sox9* expression was reduced at day 21 (Figures 4(a) and 4(b)). Levels of *Col10a1*, a marker of late hypertrophic cartilage, were upregulated at days 10 and 14 in the amygdalin treatment group (Figure 4(c)); this supported the histologic results of decreased cartilage area at day 14. Osteogenic marker genes, runt-related transcription factor 2 (*Runx2*), and *osteocalcin* (*OCN*), were also increased at days 10 and 14 in the amygdalin treatment group compared to the control group (Figures 4(d) and 4(e)). In addition, expression of *Vegf* at days 10 and 14 was increased in the amygdalin-treated group compared to the controls, indicating that amygdalin may stimulate angiogenesis during fracture healing (Figure 4(f)).

**Figure 5:**
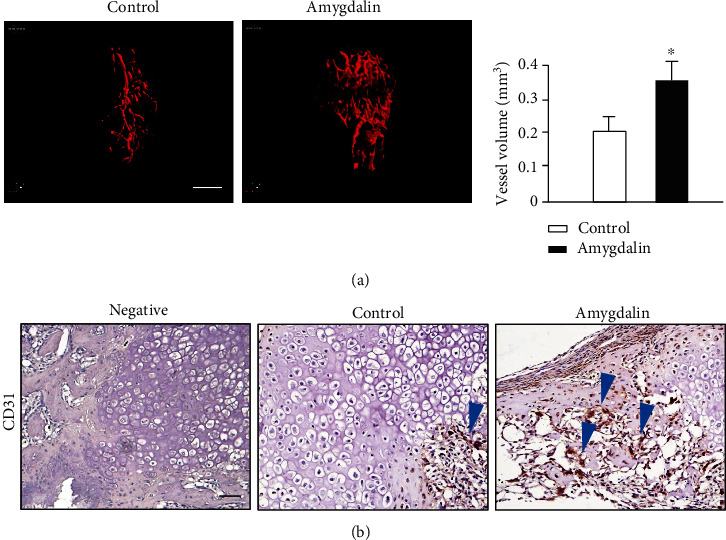
Amygdalin promoted angiogenesis in the newly formed callus around the fracture. (a) Three-dimensional images and quantification of angiography around the fractures in control and amygdalin-treated mice 10 days after surgery. Quantification shows the vessel volume. Scale bar = 1 mm. (b) CD31 protein expression was assessed by immunohistochemistry in control and amygdalin-treated groups (day 10). Blue arrows: CD31-positive cells. Scale bar = 50 *μ*m. Data are presented as mean ± standard deviation (SD). ∗*P* < 0.05, *n* = 6.

Angiographic results demonstrated that amygdalin stimulated angiogenesis and increased the vessel volume in the cartilaginous bone around the fracture callus (*P* < 0.05) (Figure 5(a)). Protein expression of the endothelial cell marker, CD31, was assessed by IHC at the callus area on day 10. Fracture callus samples from amygdalin-treated mice showed increased CD31 expression (Figure 5(b)), consistent with upregulation of the angiogenesis-related marker gene *Vegf*.

### 3.2. Amygdalin Accelerates Bone Fracture Healing through TGF-*β*/Smad Signaling of MSCs *In Vivo*

TGF-*β*/Smad signaling plays fundamental roles in bone homeostasis [25], and amygdalin has been implicated in the regulation of this pathway. To determine whether TGF-*β*/Smad signaling in MSCs is directly required for amygdalin-induced fracture healing, tibia fractures were performed on MSC-specific *Tgfβr2* conditional KO (*Tgfβr2^Gli1Cre^*) mice and Cre-negative mice.

**Figure 6:**
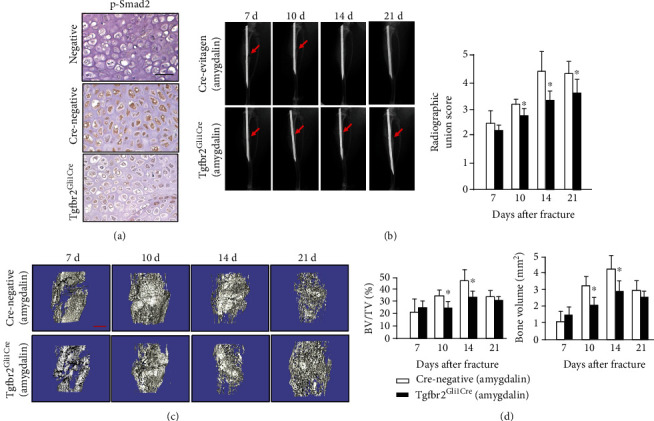
Amygdalin promoted bone fracture healing in the Cre-negative group compared with the *Tgfβr2^Gli1Cre^* group. (a) Phospho-Smad2 protein expression was assessed by immunohistochemistry in the Cre-negative and *Tgfβr2^Gli1Cre^* groups (day 7). Scale bar = 50 *μ*m. (b) X-ray results of fractures in the amygdalin-treated *Tgfβr2^Gli1Cre^* group and Cre-negative mice. Red arrows indicate fracture lines. (c, d) *μ*CT analysis of the bone volume and BV/TV (%) in callus tissues at days 7, 10, 14, and 21 postfracture in amygdalin-treated *Tgfβr2^Gli1Cre^* and Cre-negative mice. Scale bar = 500 *μ*m. Data are presented as mean ± standard deviation (SD). ∗*P* < 0.05, *n* = 6.

To determine the Tgf*β*r2 knockout efficiency in *Tgfβr2^Gli1Cre^* mice, IHC of p-Smad2 on fracture callus sections of Cre-negative mice and *Tgfbr2^Gli1Cre^* mice at day 10 was performed. The protein expression of p-Smad2 was obviously reduced in *Tgfbr2^Gli1Cre^* mice, indicating inhibition of TGF*β*/Smad pathway signaling (Figure 6(a)). Following fractures, amygdalin was administrated to all mice. X-ray results showed that fracture lines disappeared at days 14 and 21 in the Cre-negative group but were still clearly visible until day 21 in the *Tgfβr2^Gli1Cre^* group (Figure 6(b)). Furthermore, *μ*CT results indicated that amygdalin treatment increased bone volume and BV/TV around the fracture lines in Cre-negative mice compared to the *Tgfβr2^Gli1Cre^* mice, especially at days 10 and 14 (Figures 6(c) and 6(d)).

**Figure 7:**
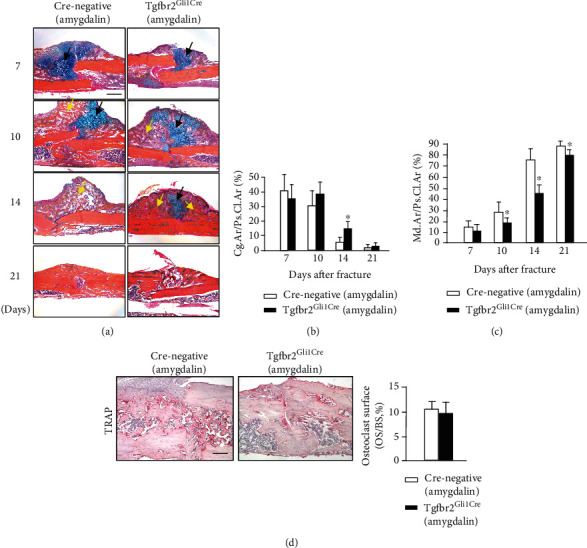
(a) Histological images of amygdalin-treated *Tgfβr2^Gli1Cre^* and Cre-negative control mice stained with Alcian blue/Hematoxylin and Eosin (ABH). Black arrows designate chondrocytes; yellow arrows highlight woven bone. Scale bar = 200 *μ*m. (b, c) The cartilage area of the callus (%, Cg.Ar/http://Ps.Cl.Ar) and newly formed woven bone (%, Md.Ar/http://Ps.Cl.Ar) were quantified at days 7, 10, 14, and 21 in amygdalin-treated *Tgfβr2^Gli1Cre^* and Cre-negative control mice. (d) Tartrate-resistant acid phosphatase (TRAP) staining and quantification of osteoclast surface per bone surface (Oc. S./BS) of sections from amygdalin-treated *Tgfβr2^Gli1-Cre^* and Cre-negative mice (day 14). Scale bar = 100 *μ*m. Data are presented as mean ± standard deviation (SD). ∗*P* < 0.05, *n* = 6.

In histological analyses of fracture callus tissues, the *Tgfβr2^Gli1Cre^* mice showed delayed fracture repair (Figure 7(a)). The cartilage area (%, Cg.Ar/http://Ps.Cl.Ar) in *Tgfβr2^Gli1Cre^* mice was significantly increased compared with controls at day 14 (Figure 7(b)). In addition, *Tgfβr2^Gli1Cre^* mice showed a weaker ability for formation of woven bone at days 10, 14, and 21 postfracture (Figure 7(c)), indicating its slower transformation from cartilage into woven bone. Even though the osteoclast number in Cre-negative mice was higher than that in *Tgfβr2^Gli1Cre^* mice, the difference was not significant (Figure 7(d)).

### 3.3. Amygdalin Promotes Migration and Differentiation of MSCs through TGF-*β*/Smad Signaling *In Vitro*

**Figure 8:**
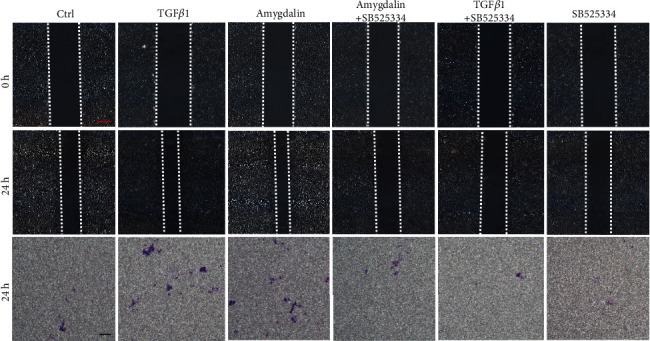
Amygdalin promoted C3H10 T1/2 cell migration through transforming growth factor- (TGF-) *β*/Smad signaling. Red scale bar = 250 *μ*m; black scale bar = 250 *μ*m.

Yu Shi et al. [21] recently found that Gli1^+^ osteogenic mesenchymal progenitors mainly facilitate normal bone formation and fracture healing. To determine if amygdalin promotes MSC migration and differentiation through TGF-*β*/Smad signaling, we conducted *in vitro* wound scratch tests and Transwell cell migration assays using C3H10 T1/2 cells. Both TGF-*β*1 (10 ng/mL) and amygdalin (10 *μ*M) treatment significantly enhanced the migration of C3H10 T1/2 cells, which could be effectively inhibited after TGF*β*/Smad signaling was blocked by treating with SB525334 (Figure 8).

**Figure 9:**
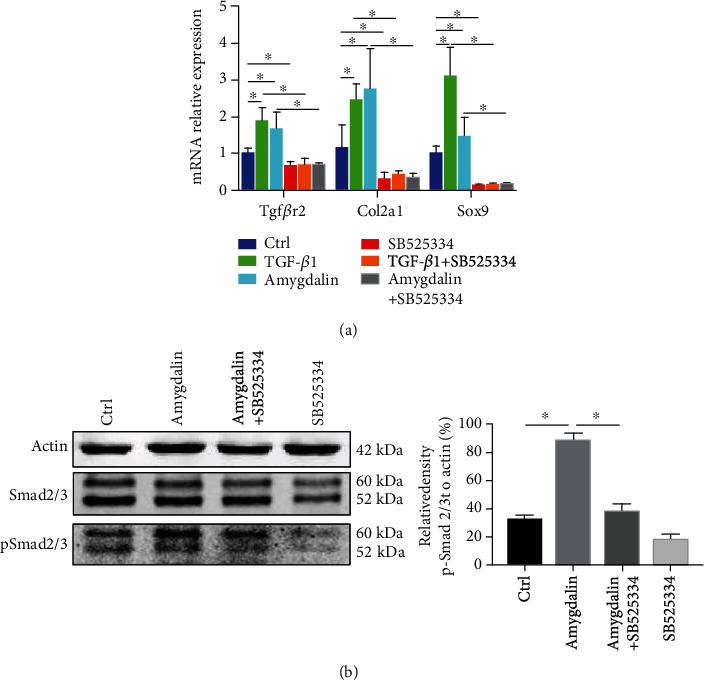
Amygdalin promoted C3H10 T1/2 cell chondrogenic gene expression through transforming growth factor- (TGF-) *β*/Smad signaling. (a) With control (Ctrl: phosphate-buffered saline), TGF-*β*1, amygdalin, and/or SB525334 treatment in C3H10 T1/2 cells, gene expressions of *Tgfβr2*, *Col2a1*, and *Sox9* were measured using qRT-PCR analysis. (b) Western blot analyses for phosphorylated SMAD2/3 were conducted on protein lysates from C3H10 T1/2 cells.

Furthermore, TGF-*β*1 and amygdalin promoted chondrogenesis in C3H10 T1/2 cells, as shown by the upregulation of chondrogenic genes, including *Sox9*, *Col2a1*, and *Tgfbr2* (Figure 9(a)). However, the effect was obviously inhibited by treatment with SB525334. In addition, phosphorylated SMAD2/3, which can regulate chondrogenesis of MSCs, was analyzed to explore the mechanism of amygdalin-induced early chondrogenic differentiation of MSCs. The p-SMAD2/3 protein expression was significantly increased in amygdalin-treated C3H10T1/2 cells, but this upregulation was inhibited in C3H10T1/2 cells treated with amygdalin plus SB525334 (Figure 9(b)). These results indicate that amygdalin can promote MSC migration and differentiation through TGF-*β*/Smad signaling.

## 4. Discussion

Bone fracture is common clinically and can cause significant patient morbidity. Several products, such as *Dalbergia sissoo*, *Peperomia pellucida*, and leaves of the *Ginkgo biloba* tree, have been investigated for potential effects on fracture repair [26–28]. The effect and molecular mechanisms of amygdalin on fracture healing, however, has not been determined. Our study demonstrated that treatment with amygdalin promotes fracture healing mainly through TGF-*β*/Smad signaling in MSCs.

Initially, two doses (0.5 and 1.0 mg/kg/day) of amygdalin were used to treat fractures in C57BL/6 mice. However, in consideration of the better effects of treatment with amygdalin at 0.5 mg/kg/day in preexperiments, we selected this lower dose for subsequent experiments. The results showed that amygdalin treatment significantly promoted endochondral ossification in the fracture callus. Radiographic data demonstrated that the radiographic union score increased after 10 days in the amygdalin group compared to the control group. *μ*CT analysis determined that amygdalin treatment significantly promoted deposition of osteoid callus, which filled the fracture gap with a higher proportion of mineralized bone, accompanied by increased bone volume. This conclusion is consistent with the results of histologic and histomorphometric analyses.

Fracture healing is a dynamic process. In mice, indirect fracture healing (also known as secondary healing) is the most common form of bone healing, usually consisting of stages of fibrocartilaginous callus formation, bony callus formation, and bone remodeling. The bone remodeling is initiated in mice from day 14 after amygdalin treatment. It is attributable to osteoclast-induced bony callus volume reduction. Radiographic union scores consist of callus formation and fracture line existence. Although there is no change of callus formation between groups, we observed fracture line and increased total radiographic union scores, which are the other indicators of improved fracture healing, in amygdalin-treated group. Fracture lines were clear at day 21 in the control group, but blurred in the amygdalin group at the same time point, indicating a better connection of cortical bone. Thus, biomechanical properties were improved with amygdalin treatment at day 21. Amygdalin treatment reduced the cartilage area and accelerated woven bone formation. However, amygdalin treatment did not increase the cartilaginous composition in early fracture callus compared to the control group. Because mineralization of the cartilaginous callus and formation of woven bone were increased by amygdalin treatment, the cartilage area of callus tissues began to decrease after day 14 compared to the control group. The transition from chondrogenesis to osteogenesis was associated with decreased gene expression of *Sox9*, a marker of chondrogenic differentiation [29], and increased expression of osteogenic markers (*Runx2* and *osteocalcin*) after amygdalin treatment. Because of further mineralization, angiogenesis and endochondral bone formation can be regulated by the later cartilage hypertrophic marker *Col10a1* [29–31]. Similarly, *Col10a1* expression was also upregulated in the amygdalin group at days 10 and 14. In addition to morphological changes, the mechanical properties of the bone could serve as an important indicator of fracture healing [32].

At the fracture site, angiogenesis and blood supply also has a direct influence on the fracture healing process. Abundant vascular distribution provides cells, growth factors, and some other constituents necessary for normal fracture healing [33, 34]. We performed angiography to examine how amygdalin impacts angiogenesis and found that it increased the volume of newly formed vessels in the cartilaginous bone. In our study, amygdalin significantly increased *Vegf* expression and CD31 protein expression in callus tissue; these findings were consistent with the results of increased angiogenesis. Previous reports revealed that angiogenesis can be induced by activation of TGF-*β* signaling [35, 36] and attenuated after inhibition of ALK5/Smad2/3 [37]. Collectively, amygdalin could promote angiogenesis during fracture healing, mainly dependent on the normal function of TGF-*β*/Smad signaling. In our study, amygdalin treatment could enhance phosphorylation of Smad2 at day 7 *in vivo*, indicating that amygdalin can upregulate TGF-*β*/Smad signaling during the fracture healing process.

During bone fracture healing, MSCs are recruited and differentiate into chondrocytes that generate cartilage extracellular matrix, which then mineralizes. Eventually, osteoblasts penetrate and promote osteogenesis [8, 38]. The fracture healing process is required for multiple cell types to repair bone structure [39]. Using lineage-tracing techniques *in vivo*, some experiments have shown that perinatal Sox9^+^, Col2^+^, or Agc1^+^ cells can produce osteoblasts and MSCs in mice [39]. Recently, however, a new type of cell, Gli1^+^ cells, were found to give rise to osteoblasts and chondrocytes in the process of bone fracture healing and to promote normal bone formation [21]. Gli1^+^ cells have been proposed to be MSCs in many bone-related studies [40, 41]. To determine whether amygdalin promotes chondrogenic and osteogenic differentiation of Gli1^+^ MSCs through TGF-*β*/Smad signaling, we tracked the fracture healing of Cre-negative and *Tgfβr2^Gli1-Cre^* mice treated with amygdalin. The MSC-specific loss of *Tgfβr2* resulted in delayed maturation and mineralization of the cartilaginous callus. Remarkably, X-ray and *μ*CT results showed delayed fracture healing in *Tgfβr2^Gli1-Cre^* mice, providing strong evidence that the stimulation of fracture healing by amygdalin depends on TGF-*β*/Smad signaling. Consistent with the *in vivo* results, both TGF-*β*1 and amygdalin-activated TGF-*β*/Smad signaling in MSCs promoted the migration of MSCs and chondrogenesis *in vitro*. Importantly, TGF-*β*/Smad inhibition by a TGF-*β*1 blocker attenuated MSC migration and the chondrogenic responses induced by TGF-*β*1 or amygdalin treatment in MSCs, confirming that amygdalin promotes facture healing in a TGF-*β*/Smad signaling-dependent mechanism.

In conclusion, this study demonstrated that amygdalin could promote the migration and differentiation of MSCs to accelerate the fracture healing process by regulating TGF-*β*/Smad signaling. These results support the use of amygdalin-based therapy for fracture healing.

## Figures and Tables

**Table 1:** Primer name and sequences for PCR analysis.

Primer name	Sequences
*β-Actin* forward	5′-GGAGATTACTGCCCTGGCTCCTA-3′
*β-Actin* reverse	5′-GACTCATCGTACTCCTGCTTGCTG-3′
*Sox9* forward	5′-GAGGCCACGGAACAGACTCA-3′
*Sox9* reverse	5′-CAGCGCCTTGAAGATAGCATT-3′
*Col2a1* forward	5′-TGGTCCTCTGGGCATCTCAGGC-3′
*Col2a1* reverse	5′-GGTGAACCTGCTGTTGCCCTCA-3′
*Col10a1* forward	5′-ACCCCAAGGACCTAAAGGAA-3′
*Col10a1* reverse	5′-CCCCAGGATACCCTGTTTTT-3′
*Runx2* forward	5′-GAGGGCACAAGTTCTATCTGGA-3′
*Runx2* reverse	5′-GGTGGTCCGCGATGATCTC-3′
*Osteocalcin* forward	5′-AGGGAGGATCAAGTCCCG-3′
*Osteocalcin* reverse	5′-GAACAGACTCCGGCGCTA-3′
*VEGF* forward	5′-GCACATAGAGAGAATGAGCTTCC-3′
*VEGF* reverse	5′-CTCCGCTCTGAACAAGGCT-3′

## Data Availability

The data in this study are available from the corresponding authors.
